# Genetic Characterization of the Poultry Red Mite (*Dermanyssus gallinae*) in Poland and a Comparison with European and Asian Isolates

**DOI:** 10.3390/pathogens11111301

**Published:** 2022-11-06

**Authors:** Sylwia Koziatek-Sadłowska, Rajmund Sokół

**Affiliations:** Department of Parasitology and Invasive Diseases, Faculty of Veterinary Medicine, University of Warmia and Mazury in Olsztyn, 10-719 Olsztyn, Poland

**Keywords:** *D. gallinae*, COI, genetic diversity

## Abstract

(1) Background: The blood-feeding mite *Dermanyssus gallinae* (De Geer 1778) continues to attract wide interest from researchers and bird breeders. The aim of this study was to evaluate the genetic diversity of *D. gallinae* populations in five commercial laying hen farms in Poland and to determine their similarity with isolates from other countries. The study involved an analysis of a fragment of the cytochrome c oxidase subunit I gene (COI). A total of 38 isolates obtained from Polish farms and 338 sequences deposited in GenBank were analyzed. (2) Results: Haplotype No. 46 was dominant (90%) in Polish isolates and was homologous with the isolates from Great Britain, the Netherlands, Belgium, Japan, and South Korea. These results are indicative of high genetic homogeneity and common ancestry of the poultry red mite and point to a common source of infestation in the examined farms. (3) Conclusions: The genetic diversity of *D. gallinae* should be further studied to promote a better understanding of how this parasite is disseminated within and between countries.

## 1. Introduction

The poultry red mite (*Dermanyssus gallinae*) (De Geer 1778) (Mesostigmata: Dermanissidae) is a broadly distributed species found on all continents except Antarctica. The poultry red mite colonizes various bird-rearing systems, regardless of production type or flock size [1]. Severe mite infestations cause a parasitic disease known as dermanyssosis. The disease compromises the well-being and health status of birds, increases mortality, and decreases laying performance and egg quality [1,2,3,4,5,6,7,8]. Its treatment is expensive, and mite infestations can generate serious economic losses in poultry farming. The losses associated with *D. gallinae* infestations in European layer farms were estimated at EUR 130 million in 2004 [9] and EUR 231 million in 2017 [10]. These data clearly indicate that red poultry mite infestations pose a serious problem in the poultry sector.

The genus *Dermanyssus* presently comprises 25 species of hematophagous mites which have been classified into two subgenera—*Dermanyssus* (*hirustus* group and *dermanyssus* group) and *Microdermanyssus* [11,12,13]. Species belonging to the first subgenus share many morphological traits and host specificity patterns. In turn, species of the *gallinae* group are difficult to distinguish based on their morphological characteristics, and colonize various bird species [12,14]. In order to identify intraspecific diversity in the genus *Dermanyssus*, the COI gene was found to be the most informative marker [12,15]. A comparative analysis of the nucleotide sequence of the COI gene revealed intraspecies variations between geographically distant populations of *D. gallinae*. The gene of 16S rRNA was informative as well as the COI gene. Research has shown that fragments of the internal transcribed spacer (ITS) were least useful marker to identify intraspecific variations within populations of *D. gallinae* [16,17,18].

It appears that genetic variation in *D. gallinae* can contribute to the plasticity in host choice and can increase the tolerance to adverse environmental conditions and selection pressures (mutations, genetic drift, natural selection and geographical isolation) [12,15]. In poultry red mites, genetic variation can result from the acquisition of resistance to a prolonged and excessive use of chemical agents [7,12,15,19].

Research into the genetic diversity of *D. gallinae* can promote a better understanding of the parasite’s population dynamics and dissemination within and between countries [20]. The resulting knowledge can be used to identify genetic markers of resistance to acaricides and to develop alternative strategies and methods to eradicate poultry red mite infestations [12,15,17]. The phylogenetic relatedness of *D. gallinae* populations in Polish poultry farms has not been fully elucidated to date. Therefore, the aim of this study was to evaluate the genetic diversity of *D. gallinae* populations in selected commercial poultry farms in Poland and to determine their similarity with isolates deposited in GenBank.

## 2. Materials and Methods

### 2.1. Dermanyssus gallinae

Female *D. gallinae* for genetic analysis were obtained from five commercial layer farms in the Polish regions of Warmia and Mazury (farms A and B), Mazovia (farms C and D), and Kuyavia-Pomerania (farm E). The mites were collected with a system of traps developed by Sokół [21]. The acquired samples were transported to a laboratory and stored at a temperature of −20 °C until analysis.

### 2.2. Isolation of Genomic DNA

Adult *D. gallinae* females were randomly selected. The mites were identified to species level based on the morphological characteristics described by Di Palma et al. [22] under a binocular stereo zoom microscope (Olympus SZ, 40× magnification). Individual mites were placed in Eppendorf tubes (1.5 mL), 300 µL of ultrapure water was added, and the contents were homogenized with the use of the Tissue Grinder Mixy Professional (NIPPON Genetics Europe, Düren, Germany). Genomic DNA was isolated with the Sherlock AX Kit (A&A Biotechnology, Gdańsk, Poland) according to the manufacturer’s protocol. The extracted DNA was suspended in 50 µL of ultrapure water. The resulting DNA was stored at a temperature of −20 °C until further analysis. The purity and quantity of the isolated DNA were checked with a spectrophotometer at a wavelength of A260/A280 and A230/A260.

### 2.3. PCR Assay

A PCR assay was conducted with the use of the StartWarm HS-PCR Mix (A&A Biotechnology, Gdańsk, Poland; catalogue No. 2017-100). The reaction mixture was composed of Taq DNA polymerase (0.1 U/µL), PCR buffer, magnesium chloride (2.5 mM), dNTPs (0.5 mM each), specific primers—forward FCOIDG (5′-CATTAATATTAACTGCACCTGACA TG-3′) and reverse RCOIDG (5′-CCCGTGGAGTGTTGAAATTCA TGA-3′) [16] or forward CO1Fyuw114 (5′-AGATCTTTAATTGAAGGGGG-3′) and reverse CO1Ryuw114 (5′-AAGATCAAAGAATCGGTGG-3′) [17] (0.5 µM each), and DNA (30–60 ng). The reaction mix had a final volume of 25 µL (12.5 µL of StartWarm HS-PCR; 1 µL of each primer, 4–5 µL of DNA, 8–14 µL of ultrapure water) or 50 µL (25 µL of StartWarm HS-PCR; 1–2 µL of each primer, 10–15 µL of DNA, 6.5–8 µL of ultrapure water). The DNA fragments amplified with the use of FCOIDG + RCOIDG and CO1Fyuw114 + CO1Ryuw114 primers had a length of 737 base pairs (bp) and 681 bp, respectively. Ultrapure nuclease-free water was added to the reaction mix in the negative control reaction. The PCR cycling conditions were as follows: initial denaturation at 95 °C for 10 min, followed by 35–40 cycles of denaturation at 95 °C for 20 s, primer annealing at 52–54 °C for 30 s, elongation at 72 °C for 90 s, and final elongation at 72 °C for 10 min. The reactions were carried out with the use of the Light Cycler Nano thermocycler (Roche, Basel, Switzerland). The PCR products were separated by electrophoresis on a 2% agarose gel with Midori Green Advance DNA Stain (NIPPON Genetics Europe, Düren, Germany). The results of the PCR assay were visualized in the Gel Doc EZ imaging system (Bio-Rad, Hercules, CA, USA) with a 100 bp molecular weight marker (Generuler 100BP DNA Ladder, Life Technologies, Carlsbad, CA, USA; catalogue No. SM0243). The products with the expected size were cut out from the agarose gel, purified with the Gel-out reagent kit (A&A Biotechnology, Gdańsk, Poland) according to the manufacturer’s protocol, and sequenced.

### 2.4. Sequencing

The obtained amplicons were sequenced by Genomed SA (Warsaw, Poland) with the use of the BigDye^®^ Terminator v3.1 Cycle Sequencing Kit (Applied Biosystems, Life Technologies).

### 2.5. Phylogenetic Analysis

#### 2.5.1. Consensus Sequences

The obtained sequences were edited in the BioEdit Sequence Alignment Editor. The quality of the resulting data was checked by analyzing the chromatograms, and ambiguous and noisy sites at the ends were removed. Low-quality reads were rejected. Antisense sequences were transcribed to sense strands using the reverse complement function. Pairs of sequences were aligned for every sample with ClustalW (to derive consensus sequences). The chromatograms corresponding to specific samples were checked to correct any sequencing errors. Non-overlapping end reads were removed. All consensus sequences were assembled with ClustalW and were trimmed to equal length. A total of 38 nucleotide sequences were obtained. The sequences were saved in a text file in FASTA format and used in further analyses.

#### 2.5.2. Homologous Sequences

Homologous sequences were obtained from the NCBI database with the use of the BLAST tool (search parameters: *Dermanyssus gallinae*; algorithm parameters; general parameters; max. target sequences: 1000). A total of 463 nucleotide sequences were exported (as of 7 August 2020) and saved in a FASTA file.

#### 2.5.3. Sequence Alignment Analysis

The generated sequences and homologous sequences were aligned with the use of ClustalW in the BioEdit environment. Sequences that were significantly shorter (less than 75% of the length of the generated sequences) were removed. All sequences were trimmed to the shortest sequence. The aligned sequences were saved in a FASTA file. A total of 376 sequences with a length of 552 bp were used in the analyses. The analyzed fragment corresponds to the nucleotide sequence from 28,218 to 28,768 of the *D. gallinae* genome (GenBank reference No. QVRM01004456.1).

#### 2.5.4. Haplotype Analysis

The sequences were collapsed to haplotypes using FaBox (https://users-birc.au.dk/palle/php/fabox/index.php (accessed on 15 August 2020)). Haplotypes are sequences that differ by at least one nucleotide or an insertion/deletion (indel) in one position. The sequences representing the studied haplotypes (one sequence per haplotype) were saved in a FASTA file and subjected to a phylogenetic analysis.

#### 2.5.5. Generation of a Phylogenetic Tree

A phylogenetic tree was generated by the neighbor-joining method (1000 bootstrap) with the use of MEGA X software. The phylogenetic tree was rooted by incorporating a homologous sequence from an external source—a fragment of the cytochrome oxidase gene of *Dermanyssus hirundinis* (FM208747.1). Evolutionary distances were estimated with the Kimura two-parameter (K2P) model and were expressed by the number of base substitutions per site. Codon positions 1, 2, 3 sequences were considered. All ambiguous positions were removed in each sequence pair (pairwise deletion option).

## 3. Results

**Figure 1:**
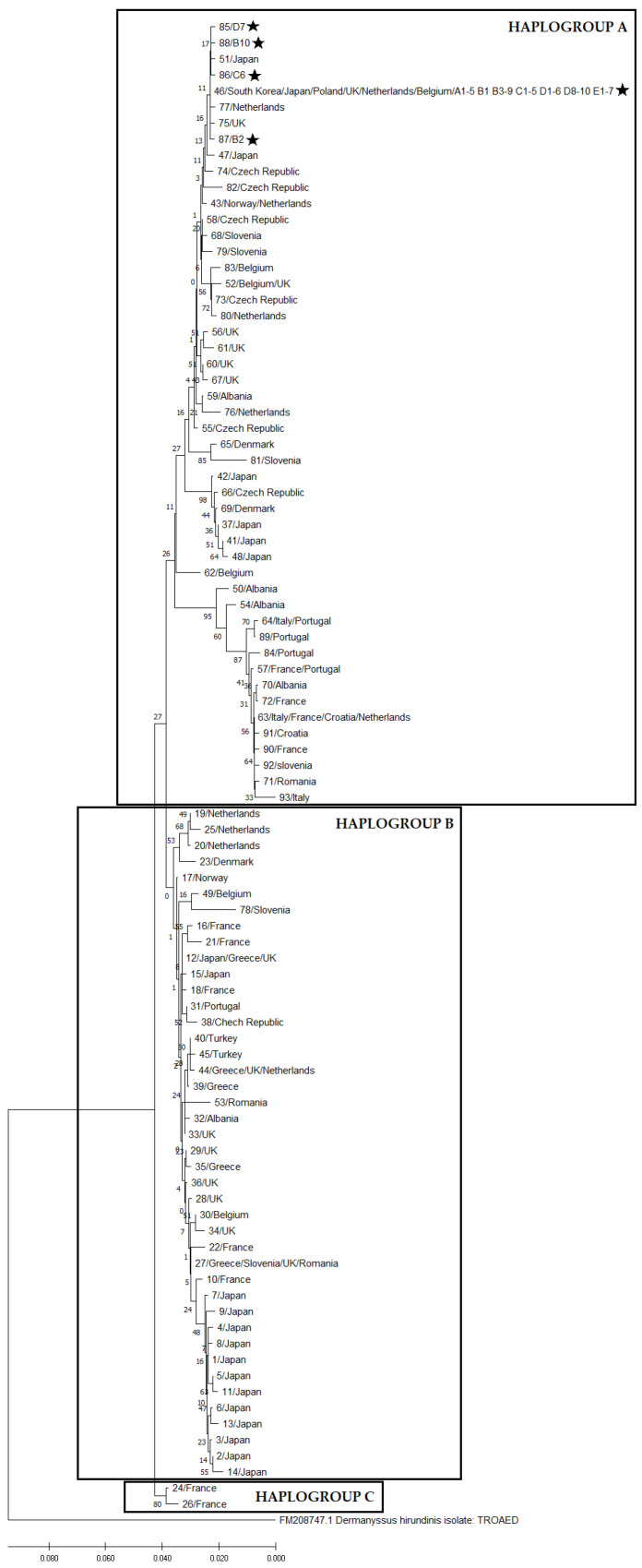
A phylogenetic tree generated by the neighbor-joining method, including haplotype numbers and countries of origin (the percentage of replicated trees, where related taxa were grouped in the bootstrap test (1000 replications), are presented next to the branches. The tree was drawn to scale, and branch lengths are given in the same units as the evolutionary distances used to infer the phylogenetic tree). (Asterisks indicate the sequences obtained in this study).

**Figure 2:**
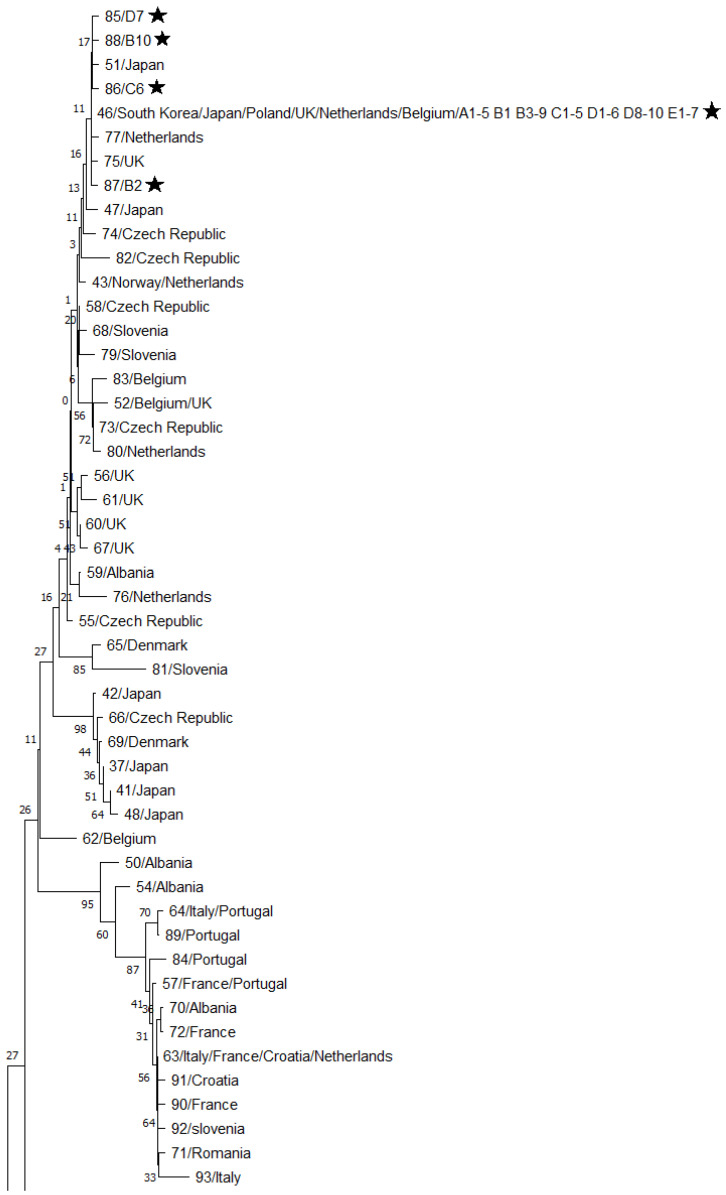
A fragment of the phylogenetic tree presenting the haplogroup A, including haplotype numbers and countries of origin. (Asterisks indicate the sequences obtained in this study).

**Figure 3:**
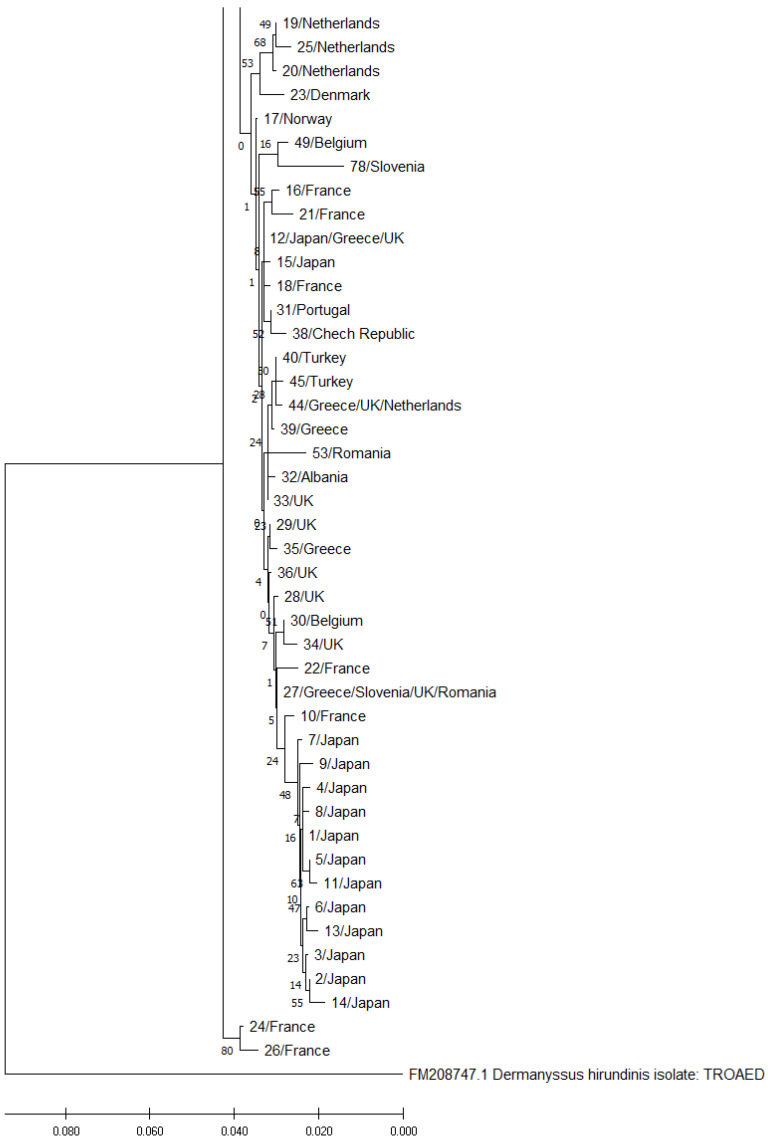
A fragment of the phylogenetic tree presenting the haplogroups B and C, including haplotype numbers and countries of origin.

A total of 93 haplotypes were identified in 376 nucleotide sequences of the 552-bp fragment of the COI gene in *D. gallinae* (including 38 sequences obtained in the study and 338 sequences obtained from the GenBank database). These haplotypes were randomly assigned numbers from 1 to 93. Haplotype numbers, GenBank reference numbers of the corresponding sequences, and countries of origin are presented in Table 1. Based on the structure of the phylogenetic tree, we divided the identified haplotypes into three haplogroups: haplogroup A (49 haplotypes), haplogroup B (42 haplotypes), and haplogroup C (2 haplotypes), which is consistent with the findings of Øines and Brännström [16] for *D. gallinae* in northern Europe. The phylogenetic tree with the haplogroups is presented in Figure 1. The haplogroups A and B are enlarged in Figure 2 and Figure 3.

The isolates from Polish poultry farms, including 5 isolates from farm A, 10 isolates from farm B, 6 isolates from farm C, 10 isolates from farm D, and 7 isolates from farm E, were assigned to 5 haplotypes belonging to the haplogroup A. These isolates were numbered 46, 85, 86, 87, and 88. Haplotype 46 contained 34 isolates (approx. 90% of all isolates). Haplotypes 85–88 were identified in single cases. The percentages of the identified haplotypes in the studied farms are presented in Table 2. *Dermanyssus gallinae* strains belonging to the haplogroups B and C were not identified in any of the samples we sequenced.

An optimal phylogenetic tree with a sum of branch lengths = 0.48525737 is presented in Figure 1.

In Polish isolates, single nucleotide substitutions were observed relative to the nucleotide sequence of the most prevalent haplotype 46. A T-to-C transition at position 550 was noted in haplotype 85. A A-to-T transversion was observed at position 552 in haplotype 86. A A-to T-transversion was noted at position 353 in haplotype 87. A G-to-A transition was observed at position 501 in haplotype 88. Insertion/deletion polymorphisms were not detected. The transversion in haplotype 87 led to the replacement of D with V in the polypeptide chain. The remaining mutations did not change the sequence of the coded polypeptide chain.

## 4. Discussion

All GenBank sequences that were homologous with the sequences of Polish isolates were considered in our genetic analysis. The reference sequences originated from various countries around the world, mostly Europe (Table 1) but also Japan and South Korea. Two lines of *D. gallinae*, classified as haplogroup A and haplogroup B, were identified as the dominant lines in the world. A third line, referred to as haplogroup C, was also identified; to date, it has been detected only in France [12]. The haplogroup C is genetically distant from haplogroups A and B, which is why haplogroup C strains are regarded as cryptic species of *D. gallinae* [16]. The haplotypes from groups A and B are ubiquitous around the world, which indicates that the parasite is transmitted between countries and continents [16,17,23]. The geographic distribution of the haplotypes in different countries suggests that haplogroup B strains have a preference for regions with a temperate climate, whereas haplogroup A strains occur in regions with a more severe climate.

The study demonstrated that *D. gallinae* haplotype 46 belonging to the haplogroup A was the most prevalent in the analyzed poultry farms (Table 2). Haplotype 46 was previously identified in Great Britain, the Netherlands, Belgium, Japan, and South Korea [16,24,23]. This haplotype was also detected by Roy et al. [12] who analyzed isolates from southern Poland. Haplotypes 85–88 were identified in individual samples, and the analysis revealed that these haplotypes probably emerged as a mutation of haplotype 46. Haplotypes 85–88, which we found in the present study, have not been identified in other countries. These observations confirm the high genetic homogeneity of *D. gallinae* populations in the analyzed Polish regions. However, based on the presented results, it cannot be concluded with sufficient certainty whether the currently occurring *D. gallinae* is a native species or whether it migrated to Poland from other European countries.

Gaweł et al. [25] reported higher levels of genetic diversity in *D. gallinae* isolates from the Polish regions of Wielkopolska, Opole, Łódź, Lubusz, and Lower Silesia. The cited authors identified four subtypes corresponding to haplogroup A and haplogroup B in the present study. The isolates from the regions of Łódź and Lubusz (10%) belonged to subtype A and were homologous to the isolates from France, the Netherlands, Denmark, and Australia. Isolates from the Wielkopolska region (83%) were homologous to the isolates from France and Poland. Two isolates from the regions of Opole and Wielkopolska were homologous to French isolates. According to the cited authors, the Polish population of *D. gallinae* is not genetically different from the populations in other European countries, which was confirmed in the current study. These data and the present findings suggest that the number of parasite transmission routes is higher in south-western than in northern Poland.

Similarly to Poland, a low genetic diversity of *D. gallinae* was also reported in Romania, Japan, and Turkey [16,23]. In contrast, *D. gallinae* populations in Greece, Great Britain, and Belgium are highly genetically diverse, and around 30 different haplotypes have been identified in these countries. Øines and Brännström [16] identified 32 haplotypes in an analysis of *D. gallinae* isolates from Sweden and Norway. Only single haplotypes were detected in most farms. These observations point to a high number of parasite transmission routes to poultry farms in Sweden and Norway. However, no haplotypes were shared between Norway and Sweden, which points to little or no exchange of *D. gallinae* strains between these countries.

The fact that females of *D. gallinae* are heterozygotic may influence the number of haplotypes. It implies that diversity may be underestimated and haplotypes cannot be unambiguously assigned in heterozygous individuals.

The results of this study indicate that *D. gallinae* populations in the examined Polish layer farms are highly genetically homogeneous and have a common ancestry, which suggests that parasitic infestations have a common source. The genetic diversity of *D. gallinae* should be studied to promote a better understanding of how this parasite is disseminated within and between countries. Understanding the spread routes of different populations of *D. gallinae* is important to advance our knowledge of their epidemiology and develop combat strategies, as different populations of *D. gallinae* may display differences in resistance to acaricides, pathogenicity and vectorial capacity [13].

## Figures and Tables

**Table 1:** Haplotypes considered in the genetic analysis, with the number of haplotypes and the country of the origin (the sequences obtained in this study are underlined).

No.	Number of Haplotypes	Sequence (GenBank ID)	Country	Ref.
1	34	MK599418	Japan	
		LC029558, LC029544, LC029537, LC029535, LC029534, LC029533, LC029531, LC029528, LC029524, LC029523, LC029522, LC029521, LC029515, LC029513, LC029512, LC029511, LC029505, LC029494, LC029492, LC029491, LC029488, LC029483, LC029481, LC029480, LC029479, LC029477, LC029476, LC029473, LC029472, LC029469, LC029468, LC029464, LC029460,	Japan	[17]
2	14	LC029542, LC029536, LC029504, LC029501, LC029499, LC029493, LC029489, LC029487, LC029482, LC029474, LC029471, LC029470, LC029466, LC029463,	Japan	[17]
3	1	LC029529	Japan	[17]
4	1	LC029467	Japan	[17]
5	2	LC029540, LC029503	Japan	[17]
6	2	LC029520, LC029462	Japan	[17]
7	2	LC029507, LC029478	Japan	[17]
8	1	LC029500	Japan	[17]
9	1	LC029458	Japan	[17]
10	1	FM208732	France	[12]
11	1	LC029553	Japan	[17]
12	13	LC029551, LC029550, LC029549, LC029548, LC029547, LC029526, LC029519, LC029518, LC029517, LC029497, LC029496,	Japan	[17]
		LR812399	Greece	[23]
		LR812378	Great Britain	[23]
13	1	LC029502	Japan	[17]
14	1	LC029465	Japan	[17]
15	1	LC029525	Japan	[17]
16	1	AM921857	France	[12]
17	1	AM921853	Norway	[12]
18	1	FM208722	France	[12]
19	1	FM207497	Netherlands	[12]
20	1	FM207495	Netherlands	[12]
21	1	AM921858	France	[12]
22	1	FM208739	France	[12]
23	1	AM921856	Denmark	[12]
24	1	AM921863	France	[12]
25	1	FM207498	Netherlands	[12]
26	1	AM921865	France	[12]
27	50	LR812452, LR812451, LR812450, LR812449, LR812448, LR812447, LR812433, LR812432, LR812431, LR812430, LR812429, LR812428, LR812427, LR812418, LR812417, LR812416, LR812415, LR812414, LR812413, LR812412, LR812411, LR812410, LR812409, LR812408, LR812407, LR812405, LR812404, LR812403, LR812402, LR812398, LR812397, LR812396, LR812395, LR812394, LR812393, LR812392, LR812391,	Greece	[23]
		LR812406	Slovenia	[23]
		LR812390, LR812373, LR812372, LR812371, LR812370, LR812369,	Great Britain	[23]
		LR812346, LR812345, LR812343, LR812342, LR812341, LR812340,	Romania	[23]
		LR812406, LR812406, LR812406	Slovenia	[23]
28	1	LR812383	Great Britain	[23]
29	1	LR812375	Great Britain	[23]
30	3	LR812339, LR812333, LR812332,	Belgium	[23]
31	3	LR812312, LR812311, LR812310	Portugal	[23]
32	2	LR812290, LR812289	Albania	[23]
33	3	LR812477, LR812377, LR812376.1	Great Britain	[23]
34	5	LR812476, LR812475, LR812474, LR81247, LR812472	Great Britain	[23]
35	1	LR812434	Greece	[23]
36	1	LR812374	Great Britain	[23]
37	1	LC029552	Japan	[17]
38	1	LR812317	Czechia	[23]
39	1	LR812420	Greece	[23]
40	5	LR812351, LR812350, LR812349, LR812348, LR812347	Turkey	[23]
41	19	LC029557, LC029556, LC029554, LC029546, LC029545, LC029541, LC029532, LC029527, LC029514, LC029510, LC029509, LC029506, LC029498, LC029490, LC029486, LC029485, LC029475, LC029459, LC029457,	Japan	[17]
42	1	LC029484	Japan	[17]
43	2	AM921852	Norway	[12]
		LR812361	Netherlands	[23]
44	27	LR812446, LR812445, LR812444, LR812443, LR812442, LR812441, LR812440, LR812439, LR812438, LR812437, LR812436, LR812435, LR812426, LR812425, LR812424, LR812423, LR812422, LR812421, LR812419, LR812401, LR812400	Netherlands	[23]
		LR812385, LR812384, LR812381, LR812380 LR812379	Great Britain	[23]
		LR812367	Netherlands	[23]
45	1	LR812352	Turkey	[23]
46	55	MN249083, MN249082, MN249080, MN249079, MN249078, MN249077, MN249076, MN249075, MN249074, MN249073, MN249072	South Korea	[24]
		LC029538, LC029516, LC029508, LC029495	Japan	[17]
		AM921854	Poland	[12]
		LR812388	Great Britain	[23]
		LR812363, LR812362	Netherlands	[23]
		LR812337, LR812336	Belgium	[23]
		A1-A5, B1, B3-9, C1-5, D 1-6, D8-10, E1-7 (OL547403-436)	Poland	
47	2	LC029543, LC029461	Japan	[17]
48	1	LC029539	Japan	[17]
49	1	LR812334	Belgium	[23]
50	1	MT232060	Albania	
51	2	LC029555, LC029530	Japan	[17]
52	2	FM208717	Belgium	[12]
		LR812470	Great Britain	[23]
53	1	LR812344	Romania	[23]
54	1	MT232059	Albania	
55	1	LR812321	Czechia	[23]
56	2	LR812468 LR812467	Great Britain	[23]
57	4	AM921864	France	[12]
		LR812307	Portugal	[23]
		LR812458, LR812455	Italy	[23]
58	2	LR812319, LR812318	Czechia	[23]
59	1	LR812288	Albania	[23]
60	8	LR812469, LR812466, LR812465, LR812464, LR812463, LR812462, LR812387, LR812386	Great Britain	[23]
61	1	LR812389	Great Britain	[23]
62	1	LR812335	Belgium	[23]
63	22	MT230034, MT230032, KY025552	Italy	
		FM208725, FM208718, FM208719, FM208733	France	[12]
		LR812301, LR812300, LR812299, LR812298,	France	[23]
		LR812296, LR812294, LR812293	Croatia	[23]
		LR812460.1, LR812459, LR812457, LR812456, LR812454, LR812453	Italy	[23]
		LR812366, LR812365	Netherlands	[23]
64	4	MT230033	Italy	
		LR812305, LR812304, LR812303	Portugal	[23]
65	4	LR812325, LR812324, LR812323, LR812326	Denmark	[23]
66	3	LR812315, LR812314, LR812313	Czechia	[23]
67	1	LR812382	Great Britain	[23]
68	1	LR812357	Slovenia	[23]
69	5	LR812331, LR812330, LR812329, LR812328, LR812327	Denmark	[23]
70	8	MT232061, LR812292, LR812291, LR812287, LR812286, LR812285, LR812284, LR812140	Albania	[23]
71	2	KX984130, KX984129	Romania	
72	1	FM208737	France	[12]
73	1	LR812320	Czechia	[23]
74	1	LR812316	Czechia	[23]
75	1	LR812471	Great Britain	[23]
76	1	LR812364	Netherlands	[23]
77	1	LR812360	Netherlands	[23]
78	1	LR812359	Slovenia	[23]
79	3	LR812358, LR812355, LR812353	Slovenia	[23]
80	1	LR812368	Netherlands	[23]
81	1	LR812356	Slovenia	[23]
82	1	LR812322	Czechia	[23]
83	1	LR812338	Belgium	[23]
84	1	LR812308	Portugal	[23]
85	1	D7 (OL547437)	Poland	
86	1	C6 (OL547438)	Poland	
87	1	B2 (OL547439)	Poland	
88	1	B10 (OL547440)	Poland	
89	1	LR812306	Portugal	[23]
90	2	LR812302, LR812297	France	[23]
91	1	LR812295	Croatia	[23]
92	1	LR812354	Slovenia	[23]
93	1	LR812461	Italy	[23]

**Table 2:** Percentages of the identified haplotypes in farms A–E.

Sampling Site (Farm)	Haplotype Number				
	46	85	86	87	88
A	100%	-	-	-	-
B	80%	-	-	10%	10%
C	83%	-	17%	-	-
D	90%	10%	-	-	-
E	100%	-	-	-	-

## Data Availability

The nucleotide sequences obtained and analyzed during the current study are available in the GenBank database (accession numbers: OL547403–OL547440).
